# Sexual Function After Transobturator Tape with or Without Concomitant Anterior Colporrhaphy in Women with Stress Urinary Incontinence and Cystocele

**DOI:** 10.3390/jcm15176905

**Published:** 2026-09-07

**Authors:** Zahide Küçük

**Affiliations:** Department of Gynecology and Obstetrics, Dr. Zahide Küçük Practice, Ankara 06530, Turkey; drzahidekucuk@outlook.com or drzahidekucuk@gmail.com; Tel.: +90-532-6290644

**Keywords:** stress urinary incontinence, cystocele, transobturator tape, anterior colporrhaphy, sexual function, Female Sexual Function Index

## Abstract

**Objectives:** To compare sexual function and other clinical outcomes after transobturator tape (TOT) alone versus TOT with concomitant anterior colporrhaphy (AC) in sexually active women with stress urinary incontinence (SUI) and asymptomatic stage ≥ 2 cystocele. **Methods:** This single-center retrospective cohort study included 173 sexually active women aged 30–50 years who underwent TOT alone (*n* = 78) or TOT with concomitant AC (TOT+AC, *n* = 95) between January 2021 and April 2025. Sexual function was assessed using the Female Sexual Function Index (FSFI) preoperatively and at 12 months postoperatively. The primary endpoint was the change in FSFI total score. Secondary outcomes included FSFI domain scores, International Consultation on Incontinence Questionnaire–Short Form (ICIQ-SF), Pelvic Organ Prolapse Symptom Score (POP-SS), Visual Analog Scale (VAS) scores, continence status, complications, and patient satisfaction. **Results:** Both groups showed significant improvements in FSFI total scores at 12 months, with a significantly greater increase in the TOT+AC group than in the TOT group (1.13 ± 0.76 vs. 0.57 ± 0.63, *p* < 0.001), corresponding to a between-group difference of 0.56 points (95% CI, 0.35–0.77). Improvements in desire (*p* = 0.004) and arousal (*p* < 0.001) were also greater in the TOT+AC group, whereas between-group differences in changes in lubrication, orgasm, satisfaction, and pain were not significant. At 12 months, the TOT+AC group had lower ICIQ-SF (2.18 ± 0.97 vs. 2.74 ± 1.11, *p* < 0.001) and POP-SS (4.02 ± 0.89 vs. 6.62 ± 1.61, *p* < 0.001) scores and higher patient satisfaction (7.71 ± 1.25 vs. 6.63 ± 1.12, *p* < 0.001). However, the magnitude of improvement in ICIQ-SF did not differ significantly between groups (*p* = 0.606). In multivariable linear regression, TOT alone remained independently associated with a smaller improvement in FSFI total score after adjustment for baseline FSFI total score and POP-Q stage (B = −0.610, 95% CI −0.842 to −0.378; *p* < 0.001). **Conclusions:** In sexually active women with SUI and asymptomatic stage ≥ 2 cystocele, concomitant AC at the time of TOT was associated with a greater improvement in overall sexual function and prolapse-related symptoms at 12 months compared with TOT alone. However, the absolute between-group difference in FSFI improvement was modest, and its clinical significance remains uncertain. Although postoperative ICIQ-SF scores were lower with TOT+AC, the magnitude of improvement in incontinence symptoms did not differ significantly between groups. These findings suggest that concomitant AC may provide additional functional benefits when individualized preoperative assessment supports combined surgery; however, the present findings do not establish specific selection criteria or support routine AC for all women with asymptomatic stage ≥ 2 cystocele. Prospective randomized studies are needed to confirm these associations and better define which patients may benefit from concomitant repair.

## 1. Introduction

SUI and pelvic organ prolapse (POP), particularly anterior compartment prolapse (cystocele), are prevalent pelvic floor disorders that can substantially impair quality of life, urinary function, and sexual well-being [1,2]. These conditions frequently coexist, and up to 50% of women undergoing surgery for SUI have concomitant POP [3]. Although the primary indication for surgery may be urinary incontinence, an accompanying anterior compartment defect may influence postoperative symptoms, patient satisfaction, and functional outcomes.

Mid-urethral sling procedures are widely established treatments for SUI because of their high efficacy and favorable safety profile [4,5]. Among these, the transobturator tape (TOT) technique provides mid-urethral support while avoiding the retropubic space. Anterior colporrhaphy (AC) can be performed concomitantly when cystocele is present; however, the optimal management of an anatomically significant but asymptomatic cystocele remains uncertain. Importantly, anatomical prolapse alone does not necessarily constitute an indication for surgical repair, particularly in women without a clinically prominent prolapse-related bulge or pressure complaint. Therefore, the decision to perform concomitant AC should be individualized according to prolapse severity, preoperative clinical findings, patient priorities, and the anticipated benefits and additional burden of combined surgery. Recent evidence suggests that concomitant midurethral sling and AC can provide favorable outcomes with acceptable complication rates in women with coexisting SUI and cystocele; however, the available evidence does not establish that all women with asymptomatic stage ≥ 2 cystocele require concomitant repair [6].

Concomitant repair may provide more comprehensive pelvic floor support and potentially reduce persistent or subsequently symptomatic prolapse [3]. Conversely, the additional procedure entails longer operative time, additional tissue dissection, and potentially greater blood loss, while its additional functional benefit remains uncertain [7,8]. These considerations are particularly relevant to sexual function. Both SUI and cystocele may adversely affect sexual well-being through coital incontinence, pelvic floor discomfort, altered body image, and dyspareunia, whereas anterior vaginal wall dissection and reconstruction may theoretically contribute to postoperative pain, dyspareunia, altered vaginal sensation, or changes in sexual response [2,8,9,10].

Mid-urethral sling surgery may improve sexual function by reducing urinary leakage and incontinence-related concerns during sexual activity [11,12]. However, whether these improvements are enhanced or attenuated when AC is performed concomitantly remains unclear. Available studies have primarily focused on anatomical success, continence, recurrence, and perioperative outcomes, while sexual function has often been evaluated only as a secondary outcome using heterogeneous instruments and follow-up periods [3,8,9]. Thus, evidence regarding the effect of concomitant TOT and AC on sexual function, particularly in sexually active women with anatomically significant but asymptomatic anterior compartment prolapse, remains limited.

Accordingly, the present study aimed to compare sexual function and other clinical outcomes between TOT alone and TOT with concomitant AC in sexually active women with SUI and asymptomatic stage ≥ 2 cystocele. The primary outcome was the change in FSFI total score from baseline to 12 months after surgery. Secondary outcomes included FSFI domains, urinary incontinence severity, prolapse-related symptom burden, patient-reported bother or pain, continence status, perioperative complications, and patient satisfaction.

## 2. Materials and Methods

### 2.1. Study Design and Ethics

This single-center, retrospective cohort study was conducted at Zahide Küçük Women’s Health and Obstetrics Clinic, Ankara, Turkey. The study included eligible women who underwent TOT surgery, either alone or in combination with concomitant AC, between January 2021 and April 2025. Accordingly, the 12-month postoperative follow-up of the last patient included in the study was completed in April 2026, before retrospective data compilation and analysis were undertaken. The study protocol was approved by the Ethics Committee of Zahide Küçük Women’s Health and Obstetrics Clinic (Approval date: 20 May 2026; decision number: TABED 1-26-2585). Following ethical approval, the completed clinical records and follow-up data were retrospectively reviewed and the anonymized study dataset was compiled for analysis. The study was conducted in accordance with the principles of the Declaration of Helsinki and its subsequent amendments.

### 2.2. Patient Selection and Inclusion Criteria

Sexually active women aged 30–50 years with SUI and concomitant asymptomatic stage ≥ 2 anterior compartment prolapse, as determined using the Pelvic Organ Prolapse Quantification (POP-Q) system, were eligible for inclusion. For the purposes of this study, “asymptomatic” anterior compartment prolapse was defined as prolapse that was not associated with a clinically prominent vaginal bulge or pressure complaint and was not the primary reason for presentation or for seeking surgical treatment. Thus, the term “asymptomatic” did not imply the complete absence of pelvic-floor-related symptoms. Patients could report nonspecific pelvic-floor symptom burden on instruments such as the POP-SS or VAS without having a clinically prominent prolapse-specific complaint. Patients who underwent TOT surgery alone or TOT with concomitant AC during the study period were retrospectively identified and classified into two groups according to the surgical procedure performed: the TOT group and the TOT+AC group.

Because of the retrospective and non-randomized design of the study, assignment to the two surgical approaches was not based on a predefined randomization protocol. The decision to perform concomitant AC was made by the operating surgeon during preoperative assessment and surgical planning, based on routine clinical practice. Specifically, the decision incorporated findings from the preoperative pelvic examination, the degree of anterior compartment descent on POP-Q assessment, the overall clinical assessment of the anterior compartment defect, and the anticipated benefit and additional surgical burden of concomitant repair. These considerations were individualized rather than applied according to a prospectively defined treatment algorithm. No single POP-Q stage alone constituted an automatic indication for concomitant AC. Accordingly, the study groups reflected the surgical management actually performed rather than prospective treatment allocation. Therefore, treatment-selection bias related to this individualized clinical decision-making process cannot be excluded.

The inclusion criteria were as follows: age 30–50 years; confirmed diagnosis of SUI based on clinical history and a positive cough stress test, with or without urodynamic confirmation [13]; asymptomatic stage ≥ 2 anterior compartment prolapse on POP-Q examination as defined above; TOT surgery performed at the study center, either alone or with concomitant AC; sexual activity, defined as at least one episode of sexual intercourse during the preceding month; completion of the FSFI both preoperatively and at the 12-month postoperative assessment; and availability of complete clinical and follow-up data.

The exclusion criteria were previous anti-incontinence surgery; predominant urge urinary incontinence or mixed urinary incontinence with a clinically significant urge component; neurogenic bladder dysfunction; history of pelvic malignancy; active urinary tract infection at the time of surgery or clinical assessment; psychiatric conditions considered likely to substantially interfere with the assessment of sexual function, such as untreated severe depression or psychosis; concomitant posterior or apical prolapse repair during the index operation; incomplete clinical or FSFI data; or loss to follow-up before the 12-month postoperative assessment.

**Figure 1:**
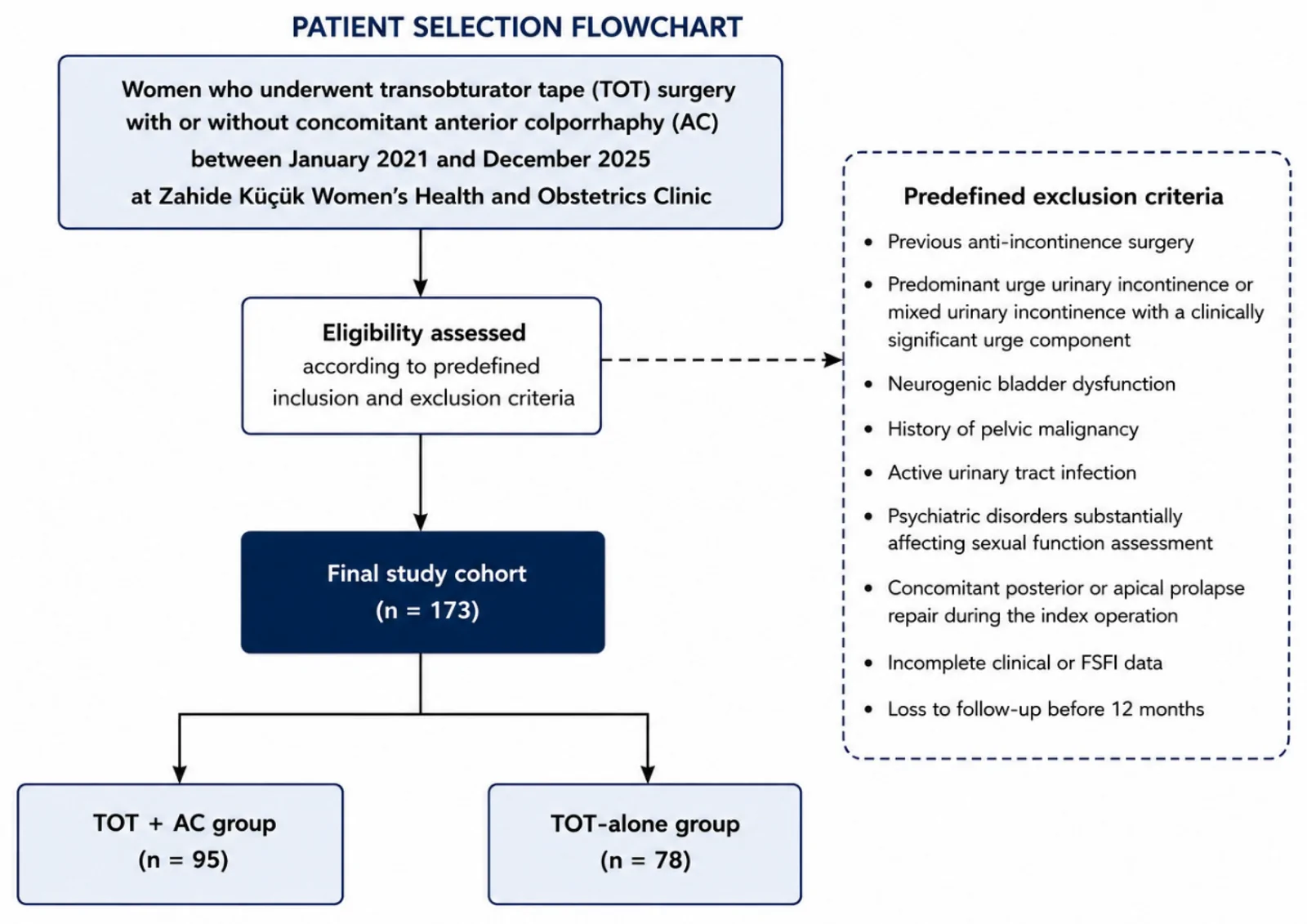
Patient selection flowchart. Flow diagram illustrating the retrospective selection of eligible patients and formation of the TOT+AC (*n* = 95) and TOT-alone (*n* = 78) study groups. AC, anterior colporrhaphy; FSFI, Female Sexual Function Index; TOT, transobturator tape.

After application of the predefined eligibility criteria, the final study cohort consisted of 173 women, including 95 who underwent TOT with concomitant AC and 78 who underwent TOT alone. The patient selection process, predefined eligibility criteria, and formation of the final study groups are summarized in Figure 1.

### 2.3. Data Collection

Data were obtained from electronic and paper medical records, operative reports, patient history forms, and questionnaires completed as part of routine clinical assessment. Following ethical approval, the anonymized dataset was compiled and analyzed without access to directly identifying patient information.

Demographic and clinical variables included age, body mass index (BMI), gravidity, parity, menopausal status, smoking status, diabetes mellitus, hypertension, vaginal estrogen use, and systemic hormone replacement therapy (HRT) use. These variables were obtained from preoperative medical records and patient history forms. The frequency of sexual intercourse per month was based on patient self-report at the preoperative assessment. The duration of SUI symptoms was obtained from the documented clinical history.

### 2.4. Surgical Procedures and Follow-Up

All patients underwent a standardized preoperative assessment that included a detailed urogynecologic history, pelvic examination with POP-Q staging, and completion of the relevant validated questionnaires. All surgical procedures were performed by the same experienced surgeon using standardized surgical techniques under spinal or general anesthesia.

The TOT procedure consisted of placement of a synthetic mid-urethral sling using the outside-in transobturator approach. In the TOT+AC group, AC was performed during the same surgical session by midline plication of the pubocervical fascia using absorbable sutures to reduce the cystocele and restore anterior compartment support.

Operative time, defined as the interval from skin incision to completion of surgical closure, estimated intraoperative blood loss, and intraoperative complications were obtained from operative records. Length of hospital stay was extracted from discharge records. Postoperative follow-up was conducted according to routine clinical practice. At the 12-month postoperative visit, patients underwent clinical reassessment and repeated administration of the study questionnaires.

### 2.5. Quality of Life and Symptom Assessment

The impact of urinary incontinence was assessed using the International Consultation on Incontinence Questionnaire–Short Form (ICIQ-SF) [14]. The ICIQ-SF is a four-item instrument with a total score ranging from 0 to 21, with higher scores indicating greater frequency, severity, and impact of urinary incontinence.

Overall symptom-related bother and pain were assessed using a 10-cm VAS, ranging from 0 (no bother/pain) to 10 (worst imaginable bother/pain). Prolapse-related symptoms were evaluated using the POP Symptom Score (POP-SS), a validated instrument assessing symptoms associated with POP [15]. The symptom reported by each patient as causing the greatest burden was also recorded.

The ICIQ-SF, POP-SS, and VAS assessments were performed preoperatively and repeated at the 12-month postoperative follow-up to evaluate changes in urinary and prolapse-related symptom burden over time.

### 2.6. Sexual Function

Sexual function was assessed using the 19-item FSFI [16], which was administered in Turkish to ensure comprehensibility for the study population. The FSFI is a 19-item, self-administered questionnaire designed to assess female sexual function during the preceding four weeks across six domains: desire (2 items), arousal (4 items), lubrication (4 items), orgasm (3 items), satisfaction (3 items), and pain (3 items).

Domain scores were calculated according to the standard FSFI scoring algorithm by multiplying individual item scores by the corresponding domain-specific factors. The total FSFI score was calculated as the sum of the six domain scores and ranged from 2 to 36, with higher scores indicating better sexual function. The FSFI was administered preoperatively and repeated at the 12-month postoperative follow-up. Changes in the FSFI total score and individual domain scores were evaluated to assess postoperative changes in sexual function.

### 2.7. Complications and Surgical Outcomes

Intraoperative and early postoperative complications, including bladder injury, mesh-related problems, and urinary retention, were identified from operative reports and postoperative follow-up records. At the 12-month follow-up, continence status was classified as cure or recurrence based on patient-reported symptoms and clinical assessment. Cure was defined as complete subjective resolution of SUI symptoms without the requirement for additional treatment.

Patient satisfaction at the 12-month postoperative assessment was also recorded and compared between the surgical groups.

### 2.8. Study Endpoints

The primary endpoint was the change in FSFI total score from the preoperative assessment to the 12-month postoperative follow-up. The principal comparison of interest was the difference in the magnitude of this change between the TOT and TOT+AC groups.

Secondary endpoints included changes in individual FSFI domain scores; changes in ICIQ-SF, POP-SS, and VAS scores from baseline to 12 months; SUI cure and recurrence rates; perioperative and postoperative complication rates; and patient satisfaction at the 12-month follow-up.

### 2.9. Statistical Analysis

All statistical analyses were performed using IBM SPSS Statistics, version 27.0 (IBM Corp., Armonk, NY, USA). All statistical tests were two-sided, and a *p*-value < 0.05 was considered statistically significant.

The distribution of continuous variables was assessed visually using histograms and Q-Q plots. Normally distributed continuous variables are presented as mean ± standard deviation, whereas non-normally distributed continuous variables are presented as median with the 25th and 75th percentiles. Categorical variables are presented as frequencies and percentages.

Between-group comparisons of normally distributed continuous variables were performed using Student’s t-test, whereas the Mann–Whitney U test was used for non-normally distributed continuous variables. Categorical variables were compared using the chi-square test, Fisher’s exact test, or Fisher–Freeman–Halton test, as appropriate. For non-normally distributed paired variables, changes between the preoperative and 12-month postoperative assessments were evaluated using the Wilcoxon signed-rank test. For normally distributed repeated measurements, two-way repeated-measures analysis of variance (ANOVA) was used to evaluate changes over time according to surgical group.

Univariable and multivariable linear regression analyses were performed to identify factors associated with the change in FSFI total score from baseline to 12 months. For the primary adjusted analysis, the multivariable model included surgical approach (TOT vs. TOT+AC) together with clinically relevant preoperative covariates selected a priori: baseline FSFI total score and POP-Q stage. Operative time and estimated blood loss were not included in the adjusted model because they represent perioperative consequences of the surgical approach rather than baseline confounders. Unstandardized regression coefficients with 95% confidence intervals (CIs) and standardized coefficients were reported. Variance inflation factors (VIFs) were calculated to assess potential multicollinearity among the variables included in the multivariable model.

## 3. Results

### 3.1. Study Population and Baseline Characteristics

A total of 173 women met the eligibility criteria and were included in the final analysis, of whom 95 underwent TOT with concomitant AC and 78 underwent TOT alone. The demographic and baseline clinical characteristics of the two groups are presented in Table 1. Median age was 43 (37–48) years in the TOT+AC group and 44 (40–48) years in the TOT group (*p* = 0.303). No significant between-group differences were observed in BMI, gravidity, parity, menopausal status, smoking status, diabetes mellitus, hypertension, vaginal estrogen use, systemic HRT use, monthly frequency of sexual intercourse, POP-Q stage distribution, or duration of SUI symptoms (all *p* > 0.05).

The distribution of anterior compartment prolapse severity was also comparable between groups. Stage 2 prolapse was present in 72 (75.79%) women in the TOT+AC group and 51 (65.38%) in the TOT group, whereas stage 3 prolapse was observed in 23 (24.21%) and 27 (34.62%) women, respectively (*p* = 0.182).

As expected with the addition of AC, operative time was significantly longer in the TOT+AC group than in the TOT group (48.05 ± 4.96 vs. 34.78 ± 7.26 min, *p* < 0.001). Estimated blood loss was also greater in the TOT+AC group (77.66 ± 20.07 vs. 49.37 ± 17.70 mL, *p* < 0.001). Median length of hospital stay was 27 (24–32) h after TOT+AC and 24 (22–32) h after TOT alone (*p* = 0.007) (Table 1).

### 3.2. Changes in Urinary and Prolapse-Related Symptoms

Preoperative VAS scores were comparable between the TOT+AC and TOT groups [4 (3–5) vs. 4 (3–5), *p* = 0.399]. At 12 months, VAS scores decreased significantly within both groups (*p* < 0.001 for both) and were lower in the TOT+AC group than in the TOT group [2 (2–3) vs. 3 (2–4), *p* = 0.003]. The reduction from baseline was also greater in the TOT+AC group [−2 (−2 to 0) vs. −1 (−2 to 0), *p* < 0.001].

ICIQ-SF scores decreased markedly from baseline to 12 months in both groups (*p* < 0.001 for both). Preoperative ICIQ-SF scores did not differ significantly between the TOT+AC and TOT groups (15.81 ± 2.59 vs. 16.18 ± 1.67, *p* = 0.279), whereas the 12-month score was significantly lower in the TOT+AC group (2.18 ± 0.97 vs. 2.74 ± 1.11, *p* < 0.001). However, the magnitude of reduction in ICIQ-SF score from baseline to 12 months was comparable between groups (−13.63 ± 2.85 vs. −13.44 ± 1.92, *p* = 0.606).

A more pronounced between-group difference was observed for prolapse-related symptoms. Baseline POP-SS scores were nearly identical between the TOT+AC and TOT groups (8.83 ± 1.48 vs. 8.78 ± 1.48, *p* = 0.827). Both groups showed significant reductions at 12 months (*p* < 0.001 for both); however, the postoperative POP-SS score was substantially lower in the TOT+AC group (4.02 ± 0.89 vs. 6.62 ± 1.61, *p* < 0.001). Correspondingly, the reduction in POP-SS was significantly greater following TOT+AC than following TOT alone (−4.81 ± 1.36 vs. −2.17 ± 1.75, *p* < 0.001) (Table 2).

### 3.3. Sexual Function Outcomes

POP-Q stage-stratified changes in FSFI total score are presented in Table 3, whereas detailed FSFI domain and total scores are presented in Table 4. At baseline, the FSFI desire score was significantly lower in the TOT+AC group than in the TOT group [3.6 (3.6–4.2) vs. 4.2 (3.6–4.2), *p* = 0.009]. Desire scores increased significantly from baseline to 12 months in both the TOT+AC (*p* < 0.001) and TOT (*p* = 0.003) groups. Importantly, the magnitude of improvement was significantly greater in the TOT+AC group (*p* = 0.004). FSFI arousal scores also improved significantly in both groups (TOT+AC, *p* < 0.001; TOT, *p* = 0.001). Although the groups did not differ significantly in absolute arousal scores at either the preoperative or 12-month assessment, the increase from baseline was significantly greater in the TOT+AC group (*p* < 0.001).

Lubrication scores increased significantly within the TOT+AC group (*p* = 0.004), whereas no significant within-group improvement was observed after TOT alone. Nevertheless, the magnitude of change in lubrication did not differ significantly between the two surgical groups, and therefore the findings did not demonstrate a differential treatment-associated improvement in this domain.

Orgasm and satisfaction domain scores improved significantly from baseline in both groups (all *p* < 0.001). Although the absolute satisfaction domain score at 12 months was higher in the TOT+AC group than in the TOT group (*p* = 0.012), the between-group difference in the magnitude of satisfaction score change was not significant. Similarly, no significant between-group difference was demonstrated for the magnitude of change in orgasm score. These findings distinguish within-group postoperative improvements from differences in improvement between the two surgical approaches.

The primary endpoint, change in FSFI total score from baseline to 12 months, demonstrated a significant difference between groups. FSFI total scores improved significantly within both surgical groups (*p* < 0.001 for both). The mean increase was 1.13 ± 0.76 in the TOT+AC group compared with 0.57 ± 0.63 in the TOT group, corresponding to a mean between-group difference of 0.56 points (95% CI, 0.35–0.77) and a standardized effect size (Cohen’s d) of 0.79 (*p* < 0.001). Thus, although improvements were observed after both surgical approaches, the magnitude of improvement in overall sexual function was greater among women who underwent concomitant AC.

In a subgroup analysis stratified by baseline POP-Q stage (Table 3), the greater improvement in FSFI total score associated with TOT+AC was observed in both prolapse-severity subgroups. Among women with stage 2 prolapse, the mean increase in FSFI total score was 1.14 ± 0.76 in the TOT+AC group and 0.58 ± 0.63 in the TOT group, corresponding to a between-group difference of 0.56 points (95% CI, 0.32–0.80; *p* < 0.001). Among women with stage 3 prolapse, the corresponding increases were 1.10 ± 0.77 and 0.55 ± 0.64, respectively, with a between-group difference of 0.55 points (95% CI, 0.16–0.94; *p* = 0.007). Thus, the observed between-group difference in FSFI improvement was not confined to women with stage 3 prolapse.

### 3.4. Continence, Complications, and Patient Satisfaction

At the 12-month assessment, the majority of women in both groups achieved cure of SUI. Cure was observed in 92 of 95 women (96.84%) in the TOT+AC group and 73 of 78 women (93.59%) in the TOT group, whereas recurrence occurred in 3 (3.16%) and 5 (6.41%) women, respectively. The difference in final continence status between the groups was not statistically significant (*p* = 0.470).

Postoperative complications were uncommon in both groups, with no evidence of a significant overall difference in complication profiles between the two surgical approaches. Thus, despite the longer operative time and greater estimated blood loss associated with concomitant AC, these perioperative differences were not accompanied by a demonstrable difference in the evaluated postoperative complication rates.

Patient-reported satisfaction at 12 months was significantly higher in the TOT+AC group than in the TOT group (7.71 ± 1.25 vs. 6.63 ± 1.12, *p* < 0.001) (Table 4).

### 3.5. Factors Associated with Improvement in Overall Sexual Function

Univariable and multivariable linear regression analyses were performed to identify factors associated with the change in FSFI total score from baseline to 12 months (Table 5). In univariable analysis, surgical approach was significantly associated with the magnitude of FSFI total score improvement. Baseline FSFI total score was also significantly associated with subsequent FSFI change, whereas POP-Q stage was not significantly associated with the outcome.

In the adjusted model, surgical approach remained independently associated with FSFI total score change after adjustment for baseline FSFI total score and POP-Q stage. Compared with TOT+AC, TOT alone was associated with a 0.610-point smaller improvement in FSFI total score (B = −0.610, 95% CI −0.842 to −0.378; standardized β = −0.401; *p* < 0.001). Baseline FSFI total score was also independently and inversely associated with subsequent improvement (B = −0.058, 95% CI −0.089 to −0.027; standardized β = −0.243; *p* < 0.001), whereas POP-Q stage 3 was not independently associated with FSFI total score change (B = −0.071, 95% CI −0.292 to 0.150; standardized β = −0.043; *p* = 0.527).

The final adjusted regression model was statistically significant [F(3,169) = 14.44, *p* < 0.001] and explained approximately 19% of the adjusted variance in FSFI total score change (adjusted R^2^ = 0.190). No substantial multicollinearity was identified in the adjusted model, with VIF values ranging from 1.04 to 1.08 (Table 5).

## 4. Discussion

The present study compared sexual, urinary, prolapse-related, perioperative, and patient-reported outcomes following TOT alone versus TOT with concomitant AC in sexually active women with SUI and asymptomatic stage ≥ 2 cystocele. Both approaches were associated with significant improvements in overall sexual function at 12 months; however, the increase in FSFI total score and improvements in desire and arousal were greater following TOT+AC. Women undergoing concomitant AC also had lower POP-SS and VAS scores and higher patient satisfaction. Although the postoperative ICIQ-SF score was lower in the TOT+AC group, the magnitude of improvement from baseline and final SUI cure rates did not differ significantly between groups. Thus, the potential additional functional benefits of concomitant AC should be considered alongside the greater perioperative burden of combined surgery.

Sexual function is an important but historically under-evaluated outcome of pelvic floor treatment [17]. Its response to surgery may vary according to the underlying anatomical disorder and surgical approach. Favorable sexual-function outcomes following vaginal wall reconstruction have been reported, indicating that reconstructive surgery does not necessarily impair sexual function [18].

The role of concomitant prolapse repair during SUI surgery remains controversial, and pelvic reconstructive surgery should be individualized according to the involved compartment, anatomical defect, symptoms, and patient characteristics [19,20,21,22]. A randomized trial in women with SUI and asymptomatic stage II cystocele reported higher SUI cure rates and fewer postoperative irritative urinary symptoms following TOT+AC than TOT alone [3]. In our cohort, although the 12-month ICIQ-SF score was lower following TOT+AC, the absolute between-group difference was small (0.56 points), and statistical significance should not be equated with established clinical significance. Moreover, the magnitude of ICIQ-SF improvement and final SUI cure rates were comparable. Accordingly, the lower postoperative ICIQ-SF score cannot be interpreted as evidence of a clinically meaningful advantage in urinary symptom improvement or clear superiority of concomitant AC for continence.

In contrast, prolapse-related symptom improvement was more pronounced following TOT+AC. Baseline POP-SS scores were comparable, whereas both the 12-month score and reduction from baseline favored TOT+AC. This is clinically plausible because AC directly addresses the anterior compartment defect. Nevertheless, because postoperative anatomical POP-Q measurements were not evaluated as a primary comparative endpoint, these findings represent patient-reported functional improvement rather than evidence of superior anatomical correction.

Mid-urethral sling surgery may improve sexual well-being by reducing coital incontinence, fear of leakage, embarrassment, and incontinence-related dyspareunia. Prospective and longer-term studies have demonstrated favorable sexual outcomes following mid-urethral sling procedures [5,11]. Conversely, concerns remain regarding vaginal wall dissection. Reductions in orgasm frequency and intensity following isolated AC have been reported and attributed to possible disruption of anterior vaginal wall neurovascular and glandular structures [8]. Differences in population and surgical context may explain the contrast with our findings, because women in our cohort had coexisting SUI and anterior compartment prolapse and underwent AC concomitantly with TOT.

The domain-level FSFI findings further emphasize this complexity. Although baseline desire was significantly lower in the TOT+AC group, this should not be interpreted as evidence that the anterior compartment prolapse was clinically symptomatic. “Asymptomatic” was defined by the absence of a clinically prominent vaginal bulge or pressure complaint attributable to the anterior compartment rather than by sexual-function scores. Sexual desire is multifactorial, and the retrospective data do not permit attribution of the baseline difference specifically to cystocele. Desire and arousal showed greater improvement following TOT+AC, whereas between-group differences in changes in lubrication, orgasm, satisfaction, and pain were not significant. The absolute between-group difference in FSFI total score improvement was modest (0.56 points; 95% CI, 0.35–0.77), despite a standardized effect size of Cohen’s d = 0.79. In the absence of an established minimal clinically important difference for FSFI total score change specifically in this surgical population, the clinical relevance of this statistically significant difference should therefore be interpreted cautiously.

Importantly, stratification by baseline POP-Q stage showed that the greater FSFI improvement associated with TOT+AC was observed in both stage 2 and stage 3 subgroups. The between-group differences were similar in magnitude (0.56 points for stage 2 and 0.55 points for stage 3), indicating that the overall finding was not confined to women with stage 3 prolapse. These subgroup findings do not, however, establish that all women with stage 2 or stage 3 cystocele should undergo concomitant AC.

Multivariable analysis provided additional support for the association. After adjustment for baseline FSFI total score and POP-Q stage, TOT alone was associated with a 0.610-point smaller FSFI improvement than TOT+AC (95% CI, −0.842 to −0.378; *p* < 0.001). Baseline FSFI was also independently associated with subsequent improvement, whereas POP-Q stage was not. The model explained approximately 19% of the variance (adjusted R^2^ = 0.190), indicating that much of the variability was attributable to factors not captured in the dataset. Therefore, the independent association with surgical approach should not be interpreted as evidence of a direct causal effect of AC.

The potential benefits of combined surgery must also be balanced against its perioperative consequences. TOT+AC was associated with longer operative time, greater estimated blood loss, and a modestly longer hospital stay, although evaluated complication rates were comparable. These findings are broadly consistent with the reported safety profile of mid-urethral sling procedures [23,24] and favorable outcomes following tension-free vaginal tape-obturator surgery [25]. The importance of individualized surgical planning and balancing expected benefits against treatment-related morbidity has also been emphasized in the broader mid-urethral sling literature [4].

The broader prolapse literature similarly demonstrates heterogeneous sexual and functional outcomes according to baseline function, prolapse compartment, surgical technique, and postoperative anatomical results [2]. Different approaches to prolapse repair have produced variable long-term outcomes [26,27,28]. A retrospective comparison of AC and TOT performed independently found no significant difference in sexual-function outcomes [29], while previous studies of concomitant prolapse repair and sling surgery further support consideration of coexisting prolapse during surgical planning [30]. Thus, anatomical cystocele alone should not automatically determine whether AC is performed.

Recent evidence further supports a nuanced interpretation. A meta-analysis by Dursun et al. demonstrated improvement in female sexual function following TOT [31]. Antosh et al. found that sexual outcomes after prolapse surgery varied according to reconstructive approach and endpoint, without evidence that pelvic reconstructive surgery uniformly worsens sexual function [32]. Similarly, Energin and Eric Horasanli reported significant short-term improvement in female sexual function following isolated anterior repair [33]. Collectively, these studies indicate that both successful treatment of SUI and restoration of pelvic support may influence postoperative sexual outcomes. Our findings extend this evidence to women with coexisting SUI and asymptomatic stage ≥ 2 anterior compartment prolapse, but the retrospective and non-randomized design precludes attribution of the greater FSFI improvement directly to AC.

**Figure 2:**
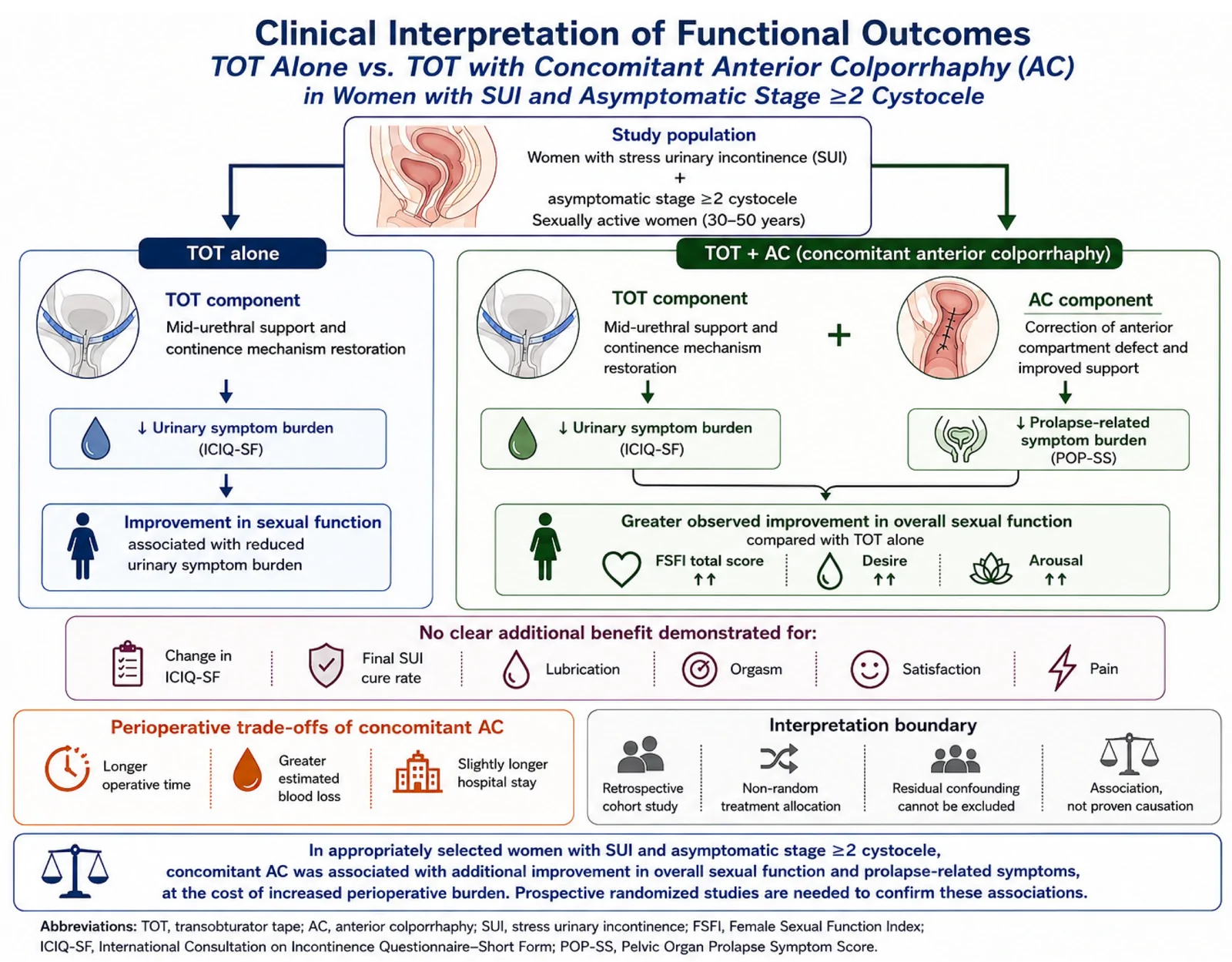
Clinical interpretation of outcomes following TOT alone versus TOT with concomitant AC. The schematic summarizes the observed functional benefits, perioperative trade-offs, and non-causal interpretation of the study findings.

The overall clinical interpretation is summarized in Figure 2. Both approaches improved urinary symptom burden and overall sexual function, whereas concomitant AC was associated with greater FSFI total score improvement, greater improvement in desire and arousal, and a greater reduction in prolapse-related symptom burden. These potential benefits should be balanced against the additional perioperative burden, and the absence of differences in ICIQ-SF improvement, final SUI cure rate, and several FSFI domains indicates that the advantage was not uniform across outcomes. Accordingly, the findings support individualized preoperative decision-making rather than routine concomitant AC for all women with asymptomatic stage ≥ 2 cystocele. Prospective randomized studies with detailed postoperative anatomical assessment and longer follow-up are required to confirm these associations.

Several limitations should be considered when interpreting the present findings. First, the retrospective, single-center design inherently introduces the possibility of selection bias and limits causal inference. In particular, the decision to perform concomitant AC was based on the operating surgeon’s clinical judgment rather than random treatment allocation or a standardized prospective algorithm. As clarified in the Methods, this individualized decision incorporated the findings of the preoperative pelvic examination, the degree of anterior compartment descent on POP-Q assessment, the overall clinical assessment of the anterior compartment defect, and the anticipated benefit and additional surgical burden of concomitant repair. Because some of these clinical considerations may also be related to subsequent functional and sexual outcomes, confounding by indication and treatment-selection bias cannot be excluded. Consequently, unmeasured clinical characteristics influencing the decision to perform AC may also have influenced postoperative outcomes. Although measured baseline characteristics were generally comparable between groups and multivariable analysis was performed for the primary endpoint, residual confounding cannot be excluded. Second, the study included only sexually active women aged 30–50 years who had complete preoperative and 12-month FSFI and follow-up data. Although these criteria were necessary for evaluation of the primary endpoint, they limit the generalizability of the findings to older women, sexually inactive women, and patients with incomplete follow-up. The requirement for complete 12-month data may also have introduced attrition-related selection bias. Third, sexual function and several other outcomes were assessed using patient-reported instruments. Although validated questionnaires provide clinically meaningful information from the patient’s perspective, sexual-function responses may be influenced by recall, social desirability, relationship characteristics, psychological factors, and cultural considerations. Partner-related factors, relationship quality, and sexual distress were not specifically assessed and could not therefore be incorporated into the analyses. Fourth, follow-up was limited to 12 months. Although this duration permits assessment of intermediate postoperative functional outcomes, it may be insufficient to identify delayed mesh-related complications, long-term recurrence of SUI or prolapse, subsequent prolapse surgery, or deterioration in sexual function over time. Longer prospective follow-up is therefore warranted. Finally, postoperative urodynamic testing was not routinely performed, and objective measures of postoperative voiding function or occult detrusor overactivity were therefore unavailable. Standardized postoperative anatomical POP-Q measurements were not consistently available in the retrospective records and therefore could not be included as a comparative anatomical outcome. This represents an important limitation, particularly because the study evaluates the potential additional value of concomitant anterior repair. Consequently, the present study cannot determine whether the greater improvement in prolapse-related symptoms following TOT+AC was accompanied by superior anatomical correction of the anterior compartment. Accordingly, the more favorable POP-SS findings after TOT+AC should be interpreted as improvements in patient-reported prolapse-related symptom burden rather than definitive evidence of superior long-term anatomical correction.

## 5. Conclusions

In sexually active women with SUI and concomitant asymptomatic stage ≥ 2 anterior compartment prolapse, both TOT alone and TOT with concomitant AC were associated with significant postoperative improvements at 12 months. However, the combined TOT+AC approach was associated with a greater improvement in overall sexual function, including greater gains in several FSFI domains, as well as greater improvement in prolapse-related symptoms, overall bother/pain, and patient satisfaction. Notably, surgical approach remained independently associated with the magnitude of FSFI total score improvement after adjustment for baseline FSFI total score and POP-Q stage, with TOT alone associated with a smaller improvement compared with TOT+AC. However, the absolute between-group difference in FSFI total score improvement was modest, and its clinical significance remains uncertain. These additional functional benefits were achieved at the expense of longer operative time, greater blood loss, and longer hospital stay, without an apparent increase in the observed complication burden.

These findings suggest that concomitant AC may represent a reasonable surgical option when an anatomically significant anterior compartment defect coexists with SUI, although the observed associations should not be interpreted as establishing clinical superiority of the combined procedure. The retrospective, non-randomized design precludes causal attribution and does not support routine concomitant AC for all women with asymptomatic cystocele. Rather, the decision should remain individualized and incorporate anterior compartment anatomy, patient priorities, expected functional benefits, and the additional perioperative burden of combined surgery. Furthermore, because standardized postoperative anatomical POP-Q data were unavailable, the study cannot determine whether the greater improvement in prolapse-related symptoms after TOT+AC was accompanied by superior anatomical correction. Prospective randomized multicenter studies with longer follow-up are warranted to determine whether the observed advantages of TOT+AC persist over time and translate into durable improvements in sexual function, anatomical outcomes, and quality of life.

## Figures and Tables

**Table 1:** Baseline demographic characteristics, clinical features, and operative outcomes of the study groups.

	Groups		
	TOT+AC (*n* = 95)	TOT (*n* = 78)	*p* (Between Groups)
Age, years	43 (37–48)	44 (40–48)	0.303 ^§^
Body mass index, kg/m^2^	28.10 ± 4.79	27.42 ± 3.79	0.295 ^†^
Gravidity	4 (3–5)	3 (3–4)	0.095 ^§^
Parity	3 (3–4)	3 (2–4)	0.095 ^§^
Menopause	19 (20.00%)	18 (23.08%)	0.761 ^#^
Smoking	15 (15.79%)	17 (21.79%)	0.415 ^#^
Diabetes mellitus	6 (6.32%)	6 (7.69%)	0.957 ^#^
Hypertension	15 (15.79%)	17 (21.79%)	0.415 ^#^
Vaginal estrogen use	1 (1.05%)	5 (6.41%)	0.092 ^‖^
Systemic HRT use	1 (1.05%)	1 (1.28%)	1.000 ^‖^
Number of intercourses, monthly	5.27 ± 1.58	5.64 ± 1.59	0.131 ^†^
POP-Q stage			
Stage 2	72 (75.79%)	51 (65.38%)	0.182 ^#^
Stage 3	23 (24.21%)	27 (34.62%)	
Duration of SUI, months	24 (15–36)	24 (12–30)	0.189 ^§^
Duration of operation, min	48.05 ± 4.96	34.78 ± 7.26	<0.001 ^†^
Blood loss, mL	77.66 ± 20.07	49.37 ± 17.70	<0.001 ^†^
Length of stay in hospital, hours	27 (24–32)	24 (22–32)	0.007 ^§^

Descriptive statistics are presented using mean ± standard deviation for normally distributed continuous variables, median (25th percentile–75th percentile) for non-normally distributed continuous variables and frequency (percentage) for categorical variables. † Student’s t test, § Mann–Whitney U test, # Chi-square test, ‖ Fisher’s exact test, AC: Anterior Colporrhaphy, HRT: Hormone Replacement Therapy, POP-Q: Pelvic Organ Prolapse Quantification, SUI: Stress Urinary Incontinence, TOT: Transobturator Tape.

**Table 2:** Preoperative and 12-month postoperative incontinence severity and clinical symptom scores.

	Groups		
	TOT+AC (*n* = 95)	TOT (*n* = 78)	*p* (Between Groups)
VAS score			
Preoperative	4 (3–5)	4 (3–5)	0.399 ^§^
Month 12	2 (2–3)	3 (2–4)	0.003 ^§^
*p* (within groups)	<0.001 ^¥^	<0.001 ^¥^	
Change ^(1)^	−2 (−2–0)	−1 (−2–0)	<0.001 ^§^
ICIQ-SF score			
Preoperative	15.81 ± 2.59	16.18 ± 1.67	0.279 ^‡^
Month 12	2.18 ± 0.97	2.74 ± 1.11	<0.001 ^‡^
*p* (within groups)	<0.001 ^‡^	<0.001 ^‡^	
Change ^(1)^	−13.63 ± 2.85	−13.44 ± 1.92	0.606 ^‡^
POP-SS			
Preoperative	8.83 ± 1.48	8.78 ± 1.48	0.827 ^‡^
Month 12	4.02 ± 0.89	6.62 ± 1.61	<0.001 ^‡^
*p* (within groups)	<0.001 ^‡^	<0.001 ^‡^	
Change ^(1)^	−4.81 ± 1.36	−2.17 ± 1.75	<0.001 ^‡^
Symptom causes most bother			
A1	0 (0.00%)	0 (0.00%)	0.848 ^¶^
A2	28 (29.47%)	18 (23.08%)	
A3	27 (28.42%)	25 (32.05%)	
A4	1 (1.05%)	0 (0.00%)	
A5	24 (25.26%)	21 (26.92%)	
A6	15 (15.79%)	14 (17.95%)	
A7	0 (0.00%)	0 (0.00%)	

Descriptive statistics are presented using mean ± standard deviation for normally distributed continuous variables, median (25th percentile–75th percentile) for non-normally distributed continuous variables and frequency (percentage) for categorical variables. ‡ Two-way repeated measures ANOVA, § Mann–Whitney U test, ¥ Wilcoxon signed ranks test, ¶ Fisher–Freeman–Halton test. ^(1)^ Difference between month 12 and preoperative assessments: negative values represent decrease and positive values represent increase. AC: Anterior Colporrhaphy, ICIQ-SF: International Consultation on Incontinence Questionnaire–Short Form, POP-SS: Pelvic Organ Prolapse Symptom Score, TOT: Transobturator Tape, VAS: Visual Analogue Scale.

**Table 3:** POP-Q stage-stratified changes in FSFI total score from baseline to 12 months according to surgical group.

POP-Q Stage	TOT+AC ΔFSFI, Mean ± SD	TOT ΔFSFI, Mean ± SD	Between-Group Difference (95% CI) *	*p*
Stage 2	1.14 ± 0.76	0.58 ± 0.63	0.56 (0.32 to 0.80)	<0.001
Stage 3	1.10 ± 0.77	0.55 ± 0.64	0.55 (0.16 to 0.94)	0.007
Overall	1.13 ± 0.76	0.57 ± 0.63	0.56 (0.35 to 0.77)	<0.001

ΔFSFI: change in Female Sexual Function Index total score from baseline to 12 months; AC: anterior colporrhaphy; POP-Q: Pelvic Organ Prolapse Quantification; TOT: transobturator tape. * Between-group difference calculated as TOT+AC minus TOT.

**Table 4:** FSFI scores, postoperative complications, and final clinical status between groups.

	Groups		
	TOT+AC (*n* = 95)	TOT (*n* = 78)	*p* (Between Groups)
FSFI Desire score			
Preoperative	3.6 (3.6–4.2)	4.2 (3.6–4.2)	0.009 ^§^
Month 12	4.2 (3.6–4.8)	4.2 (3.6–4.8)	0.967 ^§^
*p* (within groups)	<0.001 ^¥^	0.003 ^¥^	
Change ^(1)^	0.6 (0–0.6)	0 (0–0.6)	0.004 ^§^
FSFI Arousal score			
Preoperative	4.2 (3.6–4.5)	4.2 (3.9–4.2)	0.763 ^§^
Month 12	4.5 (3.9–4.5)	4.2 (3.9–4.5)	0.112 ^§^
*p* (within groups)	<0.001 ^¥^	0.001 ^¥^	
Change ^(1)^	0.3 (0–0.6)	0 (0–0.3)	<0.001 ^§^
FSFI Lubrication score			
Preoperative	4.2 (3.6–4.5)	4.2 (3.9–4.5)	0.876 ^§^
Month 12	4.2 (3.9–4.5)	4.2 (3.9–4.5)	0.645 ^§^
*p* (within groups)	0.004 ^¥^	0.309 ^¥^	
Change ^(1)^	0 (0–0.3)	0 (0–0.3)	0.254 ^§^
FSFI Orgasm score			
Preoperative	4.4 (3.6–4.4)	4.0 (3.6–4.4)	0.221 ^§^
Month 12	4.4 (3.6–4.8)	4.0 (4.0–4.4)	0.146 ^§^
*p* (within groups)	<0.001 ^¥^	<0.001 ^¥^	
Change ^(1)^	0 (0–0.4)	0 (0–0.4)	0.984 ^§^
FSFI Satisfaction score			
Preoperative	4.4 (3.6–4.4)	4.0 (3.6–4.4)	0.056 ^§^
Month 12	4.4 (4.0–4.8)	4.0 (3.6–4.4)	0.012 ^§^
*p* (within groups)	<0.001 ^¥^	<0.001 ^¥^	
Change ^(1)^	0.4 (0–0.4)	0.4 (0–0.4)	0.216 ^§^
FSFI Pain score			
Preoperative	3.43 ± 0.53	3.57 ± 0.61	0.102 ^‡^
Month 12	3.36 ± 0.47	3.47 ± 0.64	0.226 ^‡^
*p* (within groups)	0.206 ^‡^	0.065 ^‡^	
Change ^(1)^	−0.07 ± 0.48	−0.11 ± 0.53	0.603 ^‡^
FSFI Total score			
Preoperative	23.53 ± 2.34	23.79 ± 2.02	0.445 ^‡^
Month 12	24.67 ± 2.46	24.36 ± 2.06	0.382 ^‡^
*p* (within groups)	<0.001 ^‡^	<0.001 ^‡^	
Change ^(1)^	1.13 ± 0.76	0.57 ± 0.63	<0.001 ^‡^
Bladder injury	2 (2.11%)	3 (3.85%)	0.659 ^‖^
Mesh problems	4 (4.21%)	1 (1.28%)	0.380 ^‖^
Urine retention	3 (3.16%)	1 (1.28%)	0.628 ^‖^
Final status			
Cure	92 (96.84%)	73 (93.59%)	0.470 ^‖^
Recurrence	3 (3.16%)	5 (6.41%)	
Patient satisfaction	7.71 ± 1.25	6.63 ± 1.12	<0.001 ^†^

Descriptive statistics are presented using mean ± standard deviation for normally distributed continuous variables, median (25th percentile–75th percentile) for non-normally distributed continuous variables and frequency (percentage) for categorical variables. † Student’s t test, ‡ Two-way repeated measures ANOVA, § Mann–Whitney U test, ¥ Wilcoxon signed ranks test, ‖ Fisher’s exact test, ^(1)^ Difference between month 12 and preoperative assessments: negative values represent decrease and positive values represent increase. AC: anterior colporrhaphy, FSFI: Female Sexual Function Index, TOT: transobturator tape.

**Table 5:** Univariable and multivariable linear regression analyses of factors associated with change in FSFI total score from baseline to 12 months.

Variable	Univariable B (95% CI)	Standardized β	*p*	Multivariable B (95% CI)	Standardized β	*p*	VIF
Age, years	−0.013 (−0.033 to 0.007)	−0.099	0.197				
Body mass index, kg/m^2^	0.003 (−0.023 to 0.029)	0.018	0.819				
Gravidity	0.050 (−0.031 to 0.131)	0.093	0.223				
Parity	0.026 (−0.081 to 0.132)	0.036	0.636				
Menopause, Yes	−0.164 (−0.441 to 0.113)	−0.089	0.245				
Smoking, Yes	−0.310 (−0.600 to −0.020)	−0.159	0.037				
Diabetes mellitus, Yes	−0.092 (−0.541 to 0.357)	−0.031	0.686				
Hypertension, Yes	−0.045 (−0.339 to 0.249)	−0.023	0.762				
Vaginal estrogen use, Yes	−0.011 (−0.635 to 0.613)	−0.003	0.972				
Systemic HRT use, Yes	0.073 (−0.994 to 1.141)	0.010	0.892				
Number of intercourses, monthly	0.002 (−0.070 to 0.074)	0.003	0.964				
Baseline FSFI total score	−0.072 (−0.105 to −0.039)	−0.301	<0.001	−0.058 (−0.089 to −0.027)	−0.243	<0.001	1.05
POP-Q stage, Stage 3 (ref. Stage 2)	−0.086 (−0.338 to 0.165)	−0.052	0.498	−0.071 (−0.292 to 0.150)	−0.043	0.527	1.04
Duration of SUI, months	0.001 (−0.006 to 0.009)	0.023	0.763				
Duration of operation, min	0.019 (0.007 to 0.032)	0.231	0.002				
Blood loss, mL	0.006 (0.001 to 0.010)	0.175	0.021				
Length of stay in hospital, hours	0.010 (−0.009 to 0.030)	0.082	0.284				
Surgery, TOT (ref. TOT+AC)	−0.564 (−0.777 to −0.351)	−0.371	<0.001	−0.610 (−0.842 to −0.378)	−0.401	<0.001	1.08
Adjusted R^2^				0.190			
Regression model				F(3,169) = 14.44, *p* < 0.001			

Unstandardized regression coefficients (B) are presented with 95% confidence intervals (CIs). The multivariable model included surgical approach, baseline FSFI total score, and POP-Q stage. The final model was statistically significant [F(3,169) = 14.44, *p* < 0.001; adjusted R^2^ = 0.190]. AC: anterior colporrhaphy; CI: confidence interval; FSFI: Female Sexual Function Index; POP-Q: Pelvic Organ Prolapse Quantification; SUI: stress urinary incontinence; TOT: transobturator tape; VIF: variance inflation factor; HRT: hormone replacement therapy.

## Data Availability

The datasets generated and analyzed during the current study are available from the corresponding author upon reasonable request.

## Abbreviations

AC	Anterior colporrhaphy
ANOVA	Analysis of variance
BMI	Body mass index
CI	Confidence interval
FSFI	Female Sexual Function Index
HRT	Hormone replacement therapy
ICIQ-SF	International Consultation on Incontinence Questionnaire–Short Form
PISQ	Pelvic Organ Prolapse/Urinary Incontinence Sexual Questionnaire
POP	Pelvic organ prolapse
POP-Q	Pelvic Organ Prolapse Quantification
POP-SS	Pelvic Organ Prolapse Symptom Score
SUI	Stress urinary incontinence
TOT	Transobturator tape
VAS	Visual Analog Scale
VIF	Variance inflation factor
